# Evaluation of deep learning for COVID‐19 diagnosis: Impact of image dataset organization

**DOI:** 10.1002/acm2.13320

**Published:** 2021-06-23

**Authors:** Ga Young Kim, Jae Yong Kim, Chae Hyeon Kim, Sung Min Kim

**Affiliations:** ^1^ Department of Medical Biotechnology Dongguk University Goyang‐si Korea; ^2^ JLK Seoul Korea; ^3^ Department of Medical Devices Industry Dongguk University‐Seoul Seoul Korea

**Keywords:** COVID‐19, Database, deep learning, clinical application, verification

## Abstract

**Introduction:**

Coronavirus disease 2019 (COVID‐19) has spread all over the world showing high transmissibility. Many studies have proposed diverse diagnostic methods based on deep learning using chest X‐ray images focusing on performance improvement. In reviewing them, this study noticed that evaluation results might be influenced by dataset organization. Therefore, this study identified whether the high‐performance values can prove the clinical application potential.

**Methods:**

This study selected chest X‐ray image databases which have been widely applied in previous studies. One database includes images for COVID‐19, while the others consist of normal and pneumonia images. Then, the COVID‐19 classification model was designed and trained on diverse database compositions and evaluated using confusion matrix‐based metrics. Also, each database was analyzed by graphical representation methods.

**Results:**

The performance was significantly different according to dataset composition. Overall, higher performance was identified on the dataset organized with different databases for each class, compared with the dataset from same database. Also, there were significant differences in the image characteristics between different databases.

**Conclusions:**

The experimental results indicate that model may be trained based on differences of the image characteristics between databases and not on lesion features. This shows that evaluation metrics can be influenced by dataset organization, and high metric values would not directly mean the potential for clinical application. These emphasize the importance of suitable dataset organization for applying COVID‐19 diagnosis methods to real clinical sites. Radiologists should sufficiently understand about this issue as actual user of these methods.

## 1 INTRODUCTION

Coronavirus disease 2019 (COVID‐19) is an infectious disease caused by the severe acute respiratory syndrome coronavirus 2.^1^ This disease was first reported on December 2019, and it took just 30 days for the virus to spread from a single city to the entire country.^2^ Since then, the virus has rapidly spread all over the world and the World Health Organization officially declared it a pandemic on 11 March.^3^ In this urgent moment of rapidly growing cases, one of the most important ways to control its spread is early and rapid diagnosis of the disease.

The most commonly used testing method is a reverse transcription‐polymerase chain reaction (RT‐PCR). This method diagnoses COVID‐19 by detecting the presence of specific nucleic acids corresponding to SARS‐CoV‐2. However, it has low sensitivity of 60%–70% and takes a relatively long time to obtain the test result.^4^, ^5^, ^6^ RT‐PCR has been improved following the efforts of many researchers;^7^, ^8^, ^9^ nevertheless, it continues to have limitations. Furthermore, there are shortages of RT‐PCR test kits and reagents to deal with the many tests required and the test is expensive. This situation is especially more serious in countries with private health insurance systems or limited medical service systems.

The chest X‐ray image can be an alternative method for COVID‐19 diagnosis by detecting radiographic features including consolidation, ground glass opacities, and nodules.^10^, ^11^, ^12^ It has high accessibility because it is one of the most standard equipment in medical institutions and it is relatively cheap compared with other methods. Furthermore, it can be used in isolation rooms due to its portability.^6^, ^13^

Lately, a number of studies have proposed diagnosis algorithms using chest X‐ray images based on deep learning^5^, ^14^, ^15^, ^16^, ^17^ by mostly focusing on the performance improvement. For that, they organized dataset using diverse public databases and evaluated the model performance. In those experimental results, this study noticed that there was different aspect of results according to the databases and this could mean that evaluation might be influenced by the dataset organization. For example, the classification accuracy was relatively high with compositions of the specific databases. Therefore, this study realized that database should be analyzed to organize the suitable dataset and verify the clinical effectiveness of the diagnosis model for clinical adaptation to real medical system.

In deep learning, the dataset is considered as one of the important elements. For example, the size and class ratio of the dataset may impact the training process and bring positive or negative consequences.^18^, ^19^ Also, the classification results of the model can be affected by characteristics of the database. Even if the model structure is the same, a different result may be produced depending on the composition of the image dataset.

The goal of this study is to analyze the impact of the image dataset on deep learning to diagnose COVID‐19 and propose the importance of the organization of the image dataset for reliably verifying the potential for clinical application of the method. To the best of our knowledge, this is the first study that carries out a deep analysis of diverse databases related to COVID‐19. For this purpose, this study selected three kinds of chest X‐ray databases which have been widely applied in previous studies. Then, the deep learning model was designed to classify the diseases on chest X‐ray images. The classification accuracy was calculated on different compositions of the database, and they were compared.

## 2 Materials and Methods

### 2.A Chest X‐ray image datasets

The chest X‐ray image datasets were obtained from three different public databases which were used in many previous studies. One database includes images for COVID‐19 patients, and other two databases consist of normal and pneumonia images. This study will name these databases as IEEE8023, NIH X‐ray, and Chest X‐ray2017 for convenience. The details of each database were shown in Table 1.

**Table 1:** Details of X‐ray image databases.

Database name	Number of image	Description	Annotation
IEEE8023	761 images	It involves X‐ray and CT images. X‐ray dataset consists of PA, AP, APS, and lateral views. The images acquired from diverse hospitals on 26 countries.	Diseases (COVID‐19, SARS, MERS‐CoV, etc.)
NIH X‐ray	108 948 images	It consists of frontal view images (PA and AP). The images acquired from 30 805 patients with age from 0 to 95.	Normal and diseases (Eight diseases including pneumonia, pneumothorax, and cardiomegaly)
Chest X‐ray2017	5232 images	It includes 1349 normal, 2538 bacterial, and 1345 viral images. The images acquired from children.	Normal and diseases (Two diseases including bacterial and viral pneumonia)

IEEE8023 was developed by Cohen et al.^20^ and is available in Github.^21^ It contains chest X‐ray and CT images of patients with COVID‐19 and other diverse kinds of pneumonia. These images were obtained from various regions including Wuhan Jinyintan Hospital, Mount Sinai Hospital, and Myongji Hospital. In this study, only PA and AP chest X‐ray images (442 images) were selected for use as the COVID‐19 dataset.

NIH X‐ray^22^ database was provided from the National Institutes of Health, and it is also referred to as Chest X‐ray8. It consists of the frontal view X‐ray images of normal and several disease states including pneumonia, atelectasis, and cardiomegaly acquired from 30 805 patients with average age of 47. The images were labeled by using natural language processing. Chest X‐ray2017 was collected and labeled by Kermany et al.^23^ It includes a total of 5232 chest X‐ray images which were obtained from children. This study randomly selected 884 normal and 884 pneumonia images for each of two databases. These two databases can be downloaded from online.^24^, ^25^

### 2.B Classification model

**Fig. 1:**
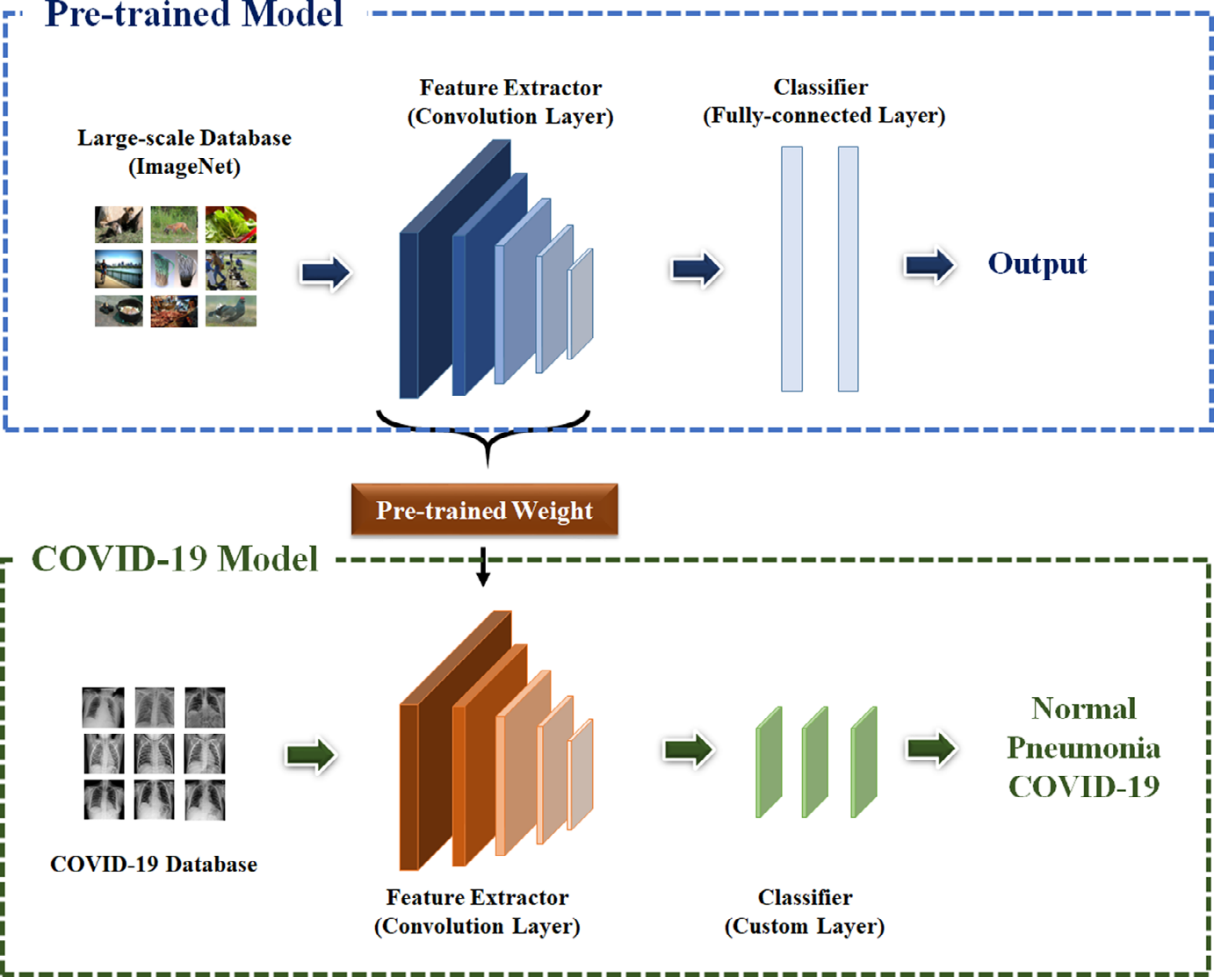
The framework of COVID‐19 diagnosis model.

This study aimed to investigate whether the organization of the image dataset is an important determinant in the ultimate clinical effectiveness of deep learning models. For this purpose, the classification model was designed as shown in Fig. 1. The proposed model consists of two main layers involving feature extractor and classifier.

In feature extraction layer, five state‐of‐the‐art models including VGG19,^26^ DenseNet121,^27^ ResNet50,^28^ InceptionV3,^29^, ^30^ and Xception^31^ were applied for extracting image features. These models are constructed with diverse types of convolution layers which are filter‐based method, and this structure can efficiently extract image features without losing spatial information. The details of each model are as follows.

**VGG19**: The VGGNet was proposed by Visual Geometry Group of Oxford University and performed very well in the ILSVRC 2014 which is the popular visual recognition challenge. It is one of the earliest try to present the impact of the depth on the model performance. It has actively applied in many studies due to its simple structure and good efficiency. VGG19 has 19 layers including 16 convolution layers and 3 fully connected layers.

**DenseNet121**: The DenseNet was designed based on the concept of the dense connectivity which means feature maps on different layers are densely connected by concatenation. This structure can mitigate the gradient vanishing problem, enhance the feature propagation, reuse the features, and improve the parameter efficiency. DenseNet121 is one of the DenseNet version including 121 layers.

**ResNet50**: The performance can be improved by increasing the depth of deep learning model. However, if the model is too deep, it may deteriorate with gradient vanishing problem. The ResNet was proposed to address this problem by using skip connection which connects the input and output of each layer. This structure can be called as residual learning and alleviates the decreasing derivative problem in backpropagation by maintaining the appropriate value. ResNet50 involves 50 layers.

**InceptionV3**: The Inception is the winner of the ILSVRC 2014. It consists of novel structure named as inception module. This module can reduce the computational expense by stacking feature sets computed from different convolutions. InceptionV3, the third version, modified inception module structure by spatially factorizing it into asymmetric convolutions. This strategy could improve the computational efficiency about 33%.

**Xception**: Xception is extreme version of the Inception. This model separately trains the cross‐channel and spatial correlation by using modified depth‐wise separable convolution. This convolution method conducts channel‐wise convolution (nxn) after point‐wise convolution (1 × 1). Also, the Xception do not include ReLU nonlinearity unlike Inception.

In this study, there were not enough chest X‐ray images, because COVID‐19 is a new disease. Although images are constantly being updated, they are still not enough to train the deep learning models. To overcome the problem which derives from an insufficient dataset, the classification models were pretrained on the large‐scale ImageNet^32^ database and the acquired weight values were reused for training the model on the chest X‐ray dataset. The weight values were fine‐tuned to better suit with the COVID‐19 classification.

To classify the X‐ray images into normal, pneumonia, or COVID‐19, the classifier was also designed based on convolution method. In most previous models, the fully connected layer was used for classification step. However, this structure converts image data into one‐dimension, and it can make model to ignore the spatial features in training process. Therefore, this study designed convolution‐based classifier to preserve the spatial structure of the image. This classifier involves three convolution layers. First and second convolution layers consist of 3 × 3 convolution, batch normalization, activation, and dropout. In the last convolution layer, 1 × 1 convolution and global average pooling were used to set the number of output to the number of classes. Also, ELU^33^ was used for activation function to efficiently train the classification model without the vanishing gradient problem.

### 2.C Performance evaluation

This study calculated confusion matrix‐based evaluation metrics to analyze the classification results according to the organization of the image datasets. In the confusion matrix, true positive (TP) and true negative (TN) means the number of positive and negative classes correctly identified as right class, respectively. False positive (FP) is the outcome where the model incorrectly predicts negative as positive, and the opposite case is false negative (FN). A total of three evaluation metrics including sensitivity, specificity, and accuracy were calculated based on these elements as shown in eqs. (1)–(3). This metrics are value between 0 and 1. And higher value indicates higher performance. The output of the model was predicted probability which is continuous value for each class. Therefore, the class with a high probability was designated as a predicted label then binary value was derived to calculate the metrics.

(1) $$\text{Sensitivity}=\;\frac{\mathrm{TP}}{TP+FN}$$

(2) $$\text{Specificity}=\;\frac{\mathrm{TN}}{TN+FP}$$

(3) $$\text{Accuracy}=\;\frac{TP+TN}{TP+TN+FP+FN}$$

This study also examined the distribution of the image datasets based on principal component analysis (PCA)^34^ and t‐distributed stochastic neighbor embedding (t‐SNE)^35^ to analyze the characteristic of the X‐ray images on each of the databases. This study identified how similar and different the image characteristics were between databases by visually representing the dataset based on these two methods. First, the original image was resized as 224 × 224 to match the shape between different images, and the matrix was converted to vector. In this study, the dimensionality of the image dataset was 50 176 (224 × 224), because the number of pixels on the image indicates dimensionality. Then, the dimensionality was reduced to two dimensions to visually represent the dataset into *X*‐*Y* axis. The resulting graph shows the characteristic distribution of image dataset.

### 2.D Experimental setup

In this study, different hyperparameters of the model were compared, and optimal conditions were set to alleviate the overfitting problem and efficiently train the model. The epoch was 100 and learning rate was 2 × 10^−6^. Adam and cross entropy were applied for optimizer and loss function. The experiment was performed on Intel Core I7‐7700K CPU and a GeForce GTX 1080 Ti GPU with TensorFlow frameworks.

## 3 Experimental results

This study proposed the importance of organizing the image dataset for reliably verifying the clinical applicability of the diagnosis method. For this purpose, this study calculated the classification results according to diverse dataset compositions. This study obtained X‐ray images from three databases which were mentioned in Section 2.1. For COVID‐19, only AP and PA X‐ray images were selected from IEEE8023, because other databases involve these two types of X‐ray images and different view features may affect the classification result. The total number of COVID‐19 dataset was 442. Also, normal and pneumonia X‐ray images were obtained from NIH X‐ray and Chest X‐ray2017 with equal number to COVID‐19 dataset to prevent class imbalance problem. However, in experiment comparing these two databases, twice as many images were applied to avoid overfitting problem. In other words, except for classification with COVID‐19, 884 images were used for each of normal and pneumonia.

To train and evaluate the model, the image dataset was separated into train and test datasets with a ratio of 0.8:0.2. Then, the train dataset was split into train and validation datasets to tune the hyperparameters. The details of train, validation, and test dataset were shown in Table 2. Also, the train dataset was augmented based on zoom, rotation, sift, and flip operators to complement the image limitations.

**Table 2:** Details of train, validation, and test dataset.

Database name	Class	Train dataset	Validation dataset	Test dataset
IEEE8023	COVID‐19	283 images	71 images	88 images
NIH X‐ray	Normal	566 images (283 images for COVID‐19 classification)	142 images (71 images for COVID‐19 classification)	176 images (88 images for COVID‐19 classification)
	Pneumonia			
Chest X‐ray2017	Normal			
	Pneumonia			

### 3.A The classification results for COVID‐19 with normal or pneumonia on different data composition

First, the five models were trained to classify the COVID‐19 and other classes. Table 3 is the binary classification results for normal and COVID‐19, and Table 4 shows the results of pneumonia and COVID‐19. In each Table, higher metrics value indicates higher performance. On NIH X‐ray, the classification accuracy was from 0.88 to 0.97 for normal and COVID‐19, and it was higher on Chest X‐ray2017 above 0.98 (Table 3). Also, the pneumonia and COVID‐19 were classified with average accuracy of 0.91 and 0.99 on the two databases, respectively (Table 4). In most models, the experimental results were higher on Chest X‐ray2017 than the NIH X‐ray.

**Table 3:** The classification results for COVID‐19 and normal on different data compositions.

Model	COVID‐19 DB	Normal DB	Sensitivity	Specificity	Accuracy
VGG19	IEEE8023	NIH X‐ray	0.96	0.98	0.97
ResNet50			0.93	0.99	0.96
DenseNet121			0.87	0.90	0.88
InceptionV3			0.93	0.97	0.95
Xception			0.92	0.98	0.95
VGG19	IEEE8023	Chest X‐ray2017	0.96	1.00	0.98
ResNet50			1.00	1.00	1.00
DenseNet121			0.98	0.98	0.98
InceptionV3			0.99	1.00	0.99
Xception			0.99	1.00	0.99

**Table 4:** The classification results for COVID‐19 and pneumonia on different data compositions.

Model	COVID‐19 DB	Pneumonia DB	Sensitivity	Specificity	Accuracy
VGG19	IEEE8023	NIH X‐ray	0.96	0.93	0.94
ResNet50			0.98	0.88	0.93
DenseNet121			0.91	0.79	0.85
InceptionV3			0.93	0.88	0.91
Xception			0.93	0.87	0.90
VGG19	IEEE8023	Chest X‐ray2017	0.98	1.00	0.98
ResNet50			1.00	1.00	1.00
DenseNet121			0.92	0.99	0.96
InceptionV3			0.99	1.00	0.99
Xception			1.00	1.00	1.00

### 3.B The classification results for normal and pneumonia on different data composition

To determine the classification results according to the dataset composition, four scenarios were designed by combining the NIH X‐ray and Chest X‐ray2017 for classification of normal and pneumonia as shown in Table 5. The experimental results demonstrated that when the different databases were combined for each class, the classification results were higher than when the same database was used for two classes. For example, in the VGG19, the classification accuracy was 0.97 when the Chest X‐ray2017 was applied for normal and pneumonia, while it was 1.00 in two scenarios with different databases for each class. Similar results were also found for ResNet50.

**Table 5:** The classification results for normal and pneumonia on different data compositions.

Model	Normal DB	Pneumonia DB	Sensitivity	Specificity	Accuracy
VGG19	NIH X‐ray	NIH X‐ray	0.75	0.79	0.77
	Chest X‐ray2017	Chest X‐ray2017	0.97	0.97	0.97
	NIH X‐ray	Chest X‐ray2017	1.00	1.00	1.00
	Chest X‐ray2017	NIH X‐ray	1.00	1.00	1.00
ResNet50	NIH X‐ray	NIH X‐ray	0.74	0.80	0.77
	Chest X‐ray2017	Chest X‐ray2017	0.97	0.99	0.98
	NIH X‐ray	Chest X‐ray2017	1.00	0.99	0.99
	Chest X‐ray2017	NIH X‐ray	1.00	1.00	1.00

### 3.C The classification results of cross‐training evaluation

This study also performed the cross‐training with different datasets to identify the robustness of the trained model and determine if the model was trained well. Cross‐training means that model was trained and tested with different databases. IEEE8023 was used either for training or testing datasets for COVID‐19. And the NIH X‐ray and Chest X‐ray2017 were applied for normal and pneumonia datasets. When the model was trained with the NIH X‐ray, Chest X‐ray2017 was used as the test dataset. The opposite procedure was also performed. Table 6 presents the classification results of cross‐training evaluation. In Table 6, the N and P on the class column indicate normal and pneumonia. The overall accuracies were under 0.75. Moreover, in some cases, the specificity was not even over 0.1. These values are significantly lower than when the models were trained and tested with same kind of database listed in Tables 1 and 2.

**Table 6:** The classification results of cross‐training evaluation on different datasets.

Model	Class	Train DB	Test DB	Sensitivity	Specificity	Accuracy
VGG19	N versus C	NIH X‐ray	Chest X‐ray2017	0.96	0.00	0.48
		Chest X‐ray2017	NIH X‐ray	0.96	0.01	0.48
	P versus C	NIH X‐ray	Chest X‐ray2017	0.98	0.09	0.53
		Chest X‐ray2017	NIH X‐ray	0.96	0.50	0.73
RestNet50	N versus C	NIH X‐ray	Chest X‐ray2017	1.00	0.00	0.50
		Chest X‐ray2017	NIH X‐ray	0.93	0.00	0.47
	P versus C	NIH X‐ray	Chest X‐ray2017	1.00	0.09	0.54
		Chest X‐ray2017	NIH X‐ray	0.98	0.22	0.60

Abbreviations: C, COVID‐19; N, Normal; P, Pneumonia.

### 3.D The visualization results of dataset

**Fig. 2:**
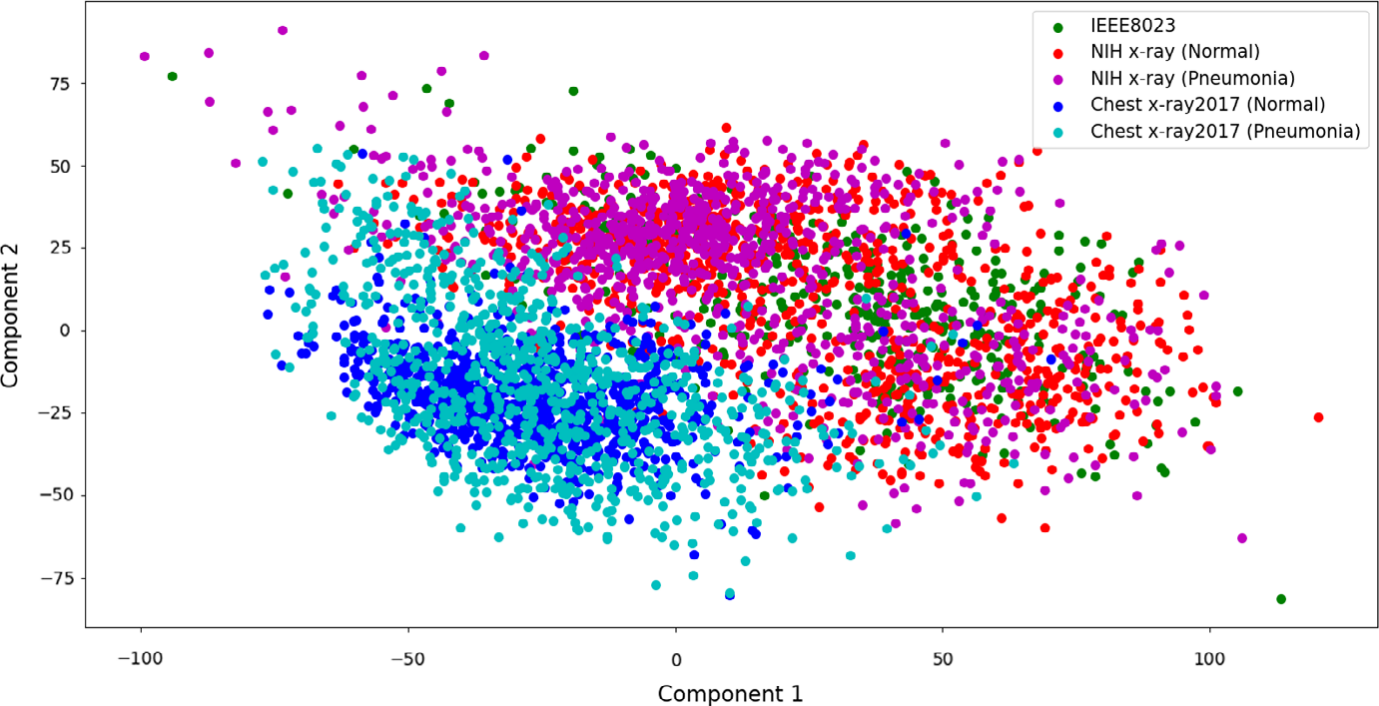
Visualization results of databases using principal component analysis (PCA).

**Fig. 3:**
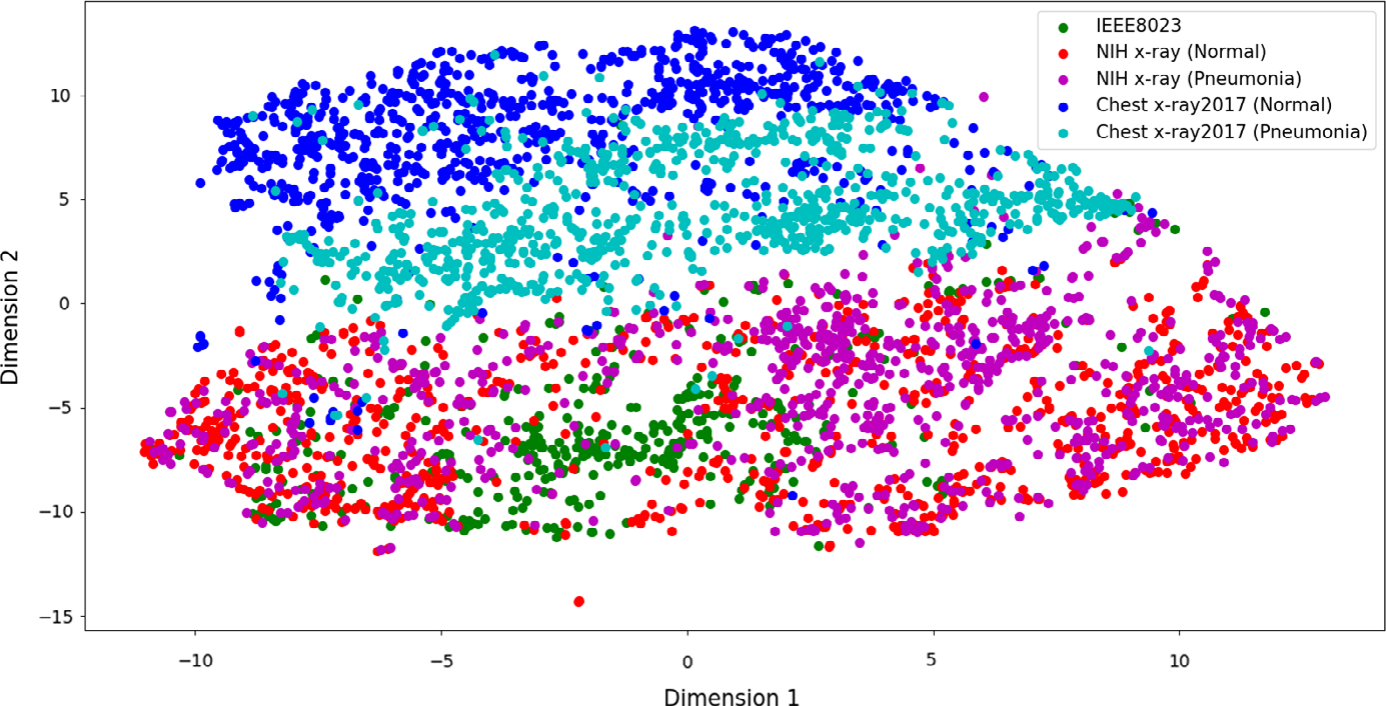
Visualization results of databases using t‐distributed stochastic neighbor embedding (t‐SNE).

To analyze the image characteristics according to the databases, the dimensionality was reduced to two dimensions. Then, the results were represented into two‐dimensional graphs. Fig. 2 is the result acquired using PCA, and Fig. 3 is for t‐SNE. The datasets were from the IEEE8023, NIH X‐ray, and Chest X‐ray2017 databases. Each of the NIH X‐ray and Chest X‐ray2017 databases was divided into two types according to the classes (normal and pneumonia). In Figs. 2 and 3, the IEEE8023 dataset was expressed as green points. The normal and pneumonia datasets of the NIH X‐ray were represented as red and magenta points. And blue and cyan were used for normal and pneumonia Chest X‐ray2017 datasets, respectively. The results show the distinct distributions according to the types of datasets.

## 4 DISCUSSION

In previous studies, there was different aspect of evaluation results according to the databases. This could mean that performance evaluation might be influenced by the dataset organization. And high evaluation values would not directly mean the potential for clinical application. Therefore, this study analyzed the impact of the image dataset on deep‐learning for COVID‐19 diagnosis and proposes the importance of dataset organization for reliably verifying the clinical applicability. For this purpose, the classification results were calculated and compared according to the diverse dataset composition.

Deep learning model was trained for binary classification of COVID‐19 and other classes. This study identified two remarkable features from the results. First, the normal and COVID‐19 was classified with higher accuracy compared with pneumonia and COVID‐19. This occurred because COVID‐19 has more distinct differences from normal than it does from pneumonia on chest X‐ray images. COVID‐19 image possess lesions like ground glass opacity and consolidation, and these can also be captured on pneumonia images.^5^ Therefore, differentiating of COVID‐19 from pneumonia may be harder than normal. Secondly, the classification results were distinctly different according to the dataset type. Overall, the models well classified on Chest X‐ray2017 with high accuracy compared with NIH X‐ray. This demonstrates that the classification performance can be affected by the dataset organization.

This study also compared the classification results for normal and pneumonia on the different dataset compositions. The classification results were relatively higher when different two databases were used for each class than organization with the same database as shown in Table 5. This implies that the model may be trained based on the different characteristics of NIH X‐ray and Chest X‐ray2017. In other words, when these two databases, respectively, used as normal and pneumonia dataset, the model may be trained to distinguish each dataset not the pathological feature. And this suggests that the model was perhaps not well trained, although it shows high performance on the evaluation metrics.

For further analysis, cross‐training was performed by using different databases for training and testing as shown in Table 6. The results were worse than using the same types of database for training and testing (Tables 3 and 4). These show that the model trained on the NIH X‐ray shows poor performance on the Chest X‐ray2017, and vice versa. These results demonstrate the low robustness of the model, and it presents that the model was trained more to be focused on the different image characteristics between databases.

This study also analyzed the image characteristic of databases by representing it into two‐dimensional graphs using PCA and t‐SNE. The resulting graphs show different characteristic distributions according to the types of datasets. The NIH X‐ray and Chest X‐ray2017 datasets show distributions distinct from each other, whereas the normal and pneumonia datasets of each of two databases show similar distributions. The distribution of the IEEE8023 dataset was relatively more similar with the NIH X‐ray datasets than the Chest X‐ray2017 datasets. These results indicate that there were significantly different image characteristics between the databases. This study concluded that the model could be trained based on these differences in image characteristics between databases and not on lesion features. Furthermore, high accuracy may not be proof that the model was well trained and shows good performance.

## 5 CONCLUSIONS

In this study, the deep learning model showed different classification results according to the dataset composition, and this illustrates that the dataset can significantly impact model performance. The goal of this study was not to distinguish which of those we tested is a good database and which is poor or to point out the quality of these popularly used databases. This study simply suggests that the dataset should be properly organized considering the developed model and object.

In the case of the databases which were applied in this study, the Chest X‐ray2017 was originally put together for developing a pneumonia diagnosis system for children under 5 yr old.^23^ It involves large numbers of X‐ray images and provides detailed information. However, this high‐quality database may not accurately fit the COVID‐19 classification problems. Because COVID‐19 classification is not for only children. The IEEE8023 database consists of X‐ray images of patients of various ages from infants to the elderly. Also, x‐ray images of each database were acquired in different environment and parameters. It is considered that these led to the differences in image characteristics between databases, and the model was may trained based on this and not the lesion features. Furthermore, NIH X‐ray was labeled based on natural language process not manual process.^22^ The accuracy is only about 90%, and this point may affect the experimental results of the X‐ray image classification.

COVID‐19 has brought many changes. It has become very difficult to move across borders, and the disease has severely influenced economies and daily life in many countries. There have been many attempts to diagnose COVID‐19 using chest X‐ray images, and this can be innovative solution for rapid and cheap diagnosis of COVID‐19. However, they still need to be verified for their clinical effectiveness to be applied to real clinical sites. And for this, more efforts are needed to obtain and organize suitable datasets for the classification of COVID‐19. Datasets should be examined before they are applied for training and evaluating the model. Information of image involving patient's gender/age, resolution, projection view, and so forth can be considered, and the different image characteristics between datasets should be analyzed to see if they are at a level that could affect the training process. The image characteristics may be calculated based on statistical approach or graphical approach. Also, as actual consumer and user of these methods, radiologists should sufficiently understand about this issue before they applying them in clinical practice.

## CONFLICT OF INTEREST

No conflicts of interest.

## Data Availability

These data were derived from the following resources available in the public domain: (https://github.com/ieee8023/covid‐chestxray‐dataset, https://nihcc.app.box.com/v/ChestXray‐NIHCC, and https://data.mendeley.com/datasets/rscbjbr9sj/2).

## References

[1] Sohrabi C , Alsafi Z , O'Neill N , et al. World Health Organization declares global emergency: a review of the 2019 novel coronavirus (COVID‐19). Int J Surg. 2020;76:71–76. 32112977 10.1016/j.ijsu.2020.02.034 PMC7105032

[2] Wu Z , McGoogan JM . Characteristics of and important lessons from the coronavirus disease 2019 (COVID‐19) outbreak in China: summary of a report of 72 314 cases from the Chinese Center for Disease Control and Prevention. JAMA. 2020;323:1239–1242. 32091533 10.1001/jama.2020.2648

[3] World Health Organization . Rolling updates on coronavirus disease (COVID‐19). https://www.who.int/emergencies/diseases/novel‐coronavirus‐2019/events‐as‐they‐happen

[4] Fang Y , Zhang H , Xie J , et al. Sensitivity of chest CT for COVID‐19: comparison to RT‐PCR. Radiology. 2020;296:E115–E117. 32073353 10.1148/radiol.2020200432 PMC7233365

[5] Ozturk T , Talo M , Yildirim EA , Baloglu UB , Yildirim O , Acharya UR . Automated detection of COVID‐19 cases using deep neural networks with X‐ray images. Comput Biol Med. 2020;121:103792. 32568675 10.1016/j.compbiomed.2020.103792 PMC7187882

[6] Wang L , Wong A .COVID‐Net: a tailored deep convolutional neural network design for detection of COVID‐19 cases from chest X‐ray images. Sci Rep. 2020;10:E115–E117. 10.1038/s41598-020-76550-z PMC7658227 33177550

[7] Chan JFW , Yip CCY , To KKW , et al. Improved molecular diagnosis of COVID‐19 by the novel, highly sensitive and specific COVID‐19‐RdRp/Hel real‐time reverse transcription‐PCR assay validated in vitro and with clinical specimens. J Clin Microbiol. 2020;58:e00310–e320. 32132196 10.1128/JCM.00310-20 PMC7180250

[8] Hirotsu Y , Mochizuki H , Omata M . Double‐quencher probes improve detection sensitivity toward Severe Acute Respiratory Syndrome Coronavirus 2 (SARS‐CoV‐2) in a reverse‐transcription polymerase chain reaction (RT‐PCR) assay. J Virol Methods. 2020;284:113926. 32650037 10.1016/j.jviromet.2020.113926 PMC7341737

[9] Zhou Y , Pei F , Ji M , et al. Sensitivity evaluation of 2019 novel coronavirus (SARS‐CoV‐2) RT‐PCR detection kits and strategy to reduce false negative. PLoS One. 2020;15:e0241469. 33206690 10.1371/journal.pone.0241469 PMC7673793

[10] Wong HYF , Lam HYS , Fong AHT , et al. Frequency and distribution of chest radiographic findings in COVID‐19 positive patients. Radiology. 2020;296:E72–E78. 32216717 10.1148/radiol.2020201160 PMC7233401

[11] Durrani M , Haq IU , Kalsoom U , Yousaf A . Chest X‐rays findings in COVID 19 patients at a University Teaching Hospital‐A descriptive study. Pak. J Med Sci. 2020;36:S22–S26. 10.12669/pjms.36.COVID19-S4.2778 PMC7306947 32582309

[12] Jacobi A , Chung M , Bernheim A , Eber C . Portable chest X‐ray in coronavirus disease‐19 (COVID‐19): a pictorial review. Clin Imaging. 2020;64:35–42. 32302927 10.1016/j.clinimag.2020.04.001 PMC7141645

[13] Schiaffino S , Tritella S , Cozzi A , et al. Diagnostic performance of chest X‐ray for COVID‐19 pneumonia during the SARS‐CoV‐2 pandemic in Lombardy, Italy. J Thorac Imaging. 2020;35:W105–W106. 32404797 10.1097/RTI.0000000000000533

[14] Khan AI , Shah JL , Bhat MM . Coronet: a deep neural network for detection and diagnosis of COVID‐19 from chest X‐ray images. Comput Methods Programs Biomed. 2020;196:105581. 32534344 10.1016/j.cmpb.2020.105581 PMC7274128

[15] Narin A , Kaya C , Pamuk Z . Automatic detection of coronavirus disease (Covid‐19) using X‐ray images and deep convolutional neural networks. arXiv:2003.10849. 2020. 10.1007/s10044-021-00984-y PMC8106971 33994847

[16] Ucar F , Korkmaz D . COVIDiagnosis‐net: deep Bayes‐squeezenet based diagnostic of the coronavirus disease 2019 (COVID‐19) from x‐ray images. Med Hypotheses. 2020;140:109761. 32344309 10.1016/j.mehy.2020.109761 PMC7179515

[17] Loey M , Smarandache F , Khalifa NEM . Within the lack of chest COVID‐19 x‐ray dataset: a novel detection model based on GAN and deep transfer learning. Symmetry. 2020;12:651.

[18] Barbedo JGA . Impact of dataset size and variety on the effectiveness of deep learning and transfer learning for plant disease classification. Comput Electron Agric. 2018;153:46–53.

[19] Johnson JM , Khoshgoftaar TM . Survey on deep learning with class imbalance. J Big Data. 2019;6:27.

[20] Cohen JP , Morrison P , Dao L , Roth K , Duong TQ , Ghassemi M . Covid‐19 image data collection: prospective predictions are the future. arXiv:2006.11988. 2020.

[21] https://github.com/ieee8023/covid‐chestxray‐dataset.

[22] Wang X , Peng Y , Lu L , Lu Z , Bagheri M , Summers RM . ChestX‐ray8: hospital‐scale chest X‐ray database and benchmarks on weakly‐supervised classification and localization of common thorax diseases. 2017.In: IEEE conference on computer vision and pattern recognition.

[23] Kermany DS , Goldbaum M , Cai W , et al. Identifying medical diagnoses and treatable diseases by image‐based deep learning. Cell. 2018;172:1122–1131. 29474911 10.1016/j.cell.2018.02.010

[24] The National Institutes of Health Clinical Center . https://nihcc.app.box.com/v/ChestXray‐NIHCC

[25] University of California San Diego . https://data.mendeley.com/datasets/rscbjbr9sj/2

[26] Simonyan K , Zisserman A . Very deep convolutional networks for large‐scale image recognition. arXiv:1409.1556. 2014.

[27] Huang G , Liu Z , Van Der Maaten L , Weinberger KQ . Densely connected convolutional networks. 2017.In: IEEE conference on computer vision and pattern recognition.

[28] He K , Zhang X , Ren S , Sun J . Deep residual learning for image recognition. 2016. In: IEEE conference on computer vision and pattern recognition.

[29] Szegedy C , Liu W , Jia Y , et al. Going deeper with convolutions. 2015. In: IEEE conference on computer vision and pattern recognition.

[30] Szegedy C , Vanhoucke V , Ioffe S , Shlens J , Wojna Z . Rethinking the inception architecture for computer vision. 2016. In: IEEE conference on computer vision and pattern recognition.

[31] Chollet F . Xception: deep learning with depthwise separable convolutions. 2017. In: IEEE conference on computer vision and pattern recognition.

[32] Deng J , Dong W , Socher R , Li LJ , Li K , Fei‐Fei L . Imagenet: a large‐scale hierarchical image database. 2009.In: IEEE conference on computer vision and pattern recognition.

[33] Clevert DA , Unterthiner T , Hochreiter S . Fast and accurate deep network learning by exponential linear units (elus). arXiv:1511.07289. 2015.

[34] Wold S , Esbensen K , Geladi P . Principal component analysis. Chemometr Intell Lab Syst. 1987;2:37–52.

[35] Maaten LVD , Hinton G . Visualizing data using t‐SNE. J Mach Learn Res. 2008;9:2579–2605.

